# Evaluation of microcurrent as an adjunct to donepezil therapy in an Alzheimer’s disease mouse model: a pilot study

**DOI:** 10.3389/fnagi.2025.1689593

**Published:** 2025-11-27

**Authors:** Eun Ho Kim, Yoon-Jin Lee, Yong Suk Moon, Oh Dae Kwon, Dong Rak Kwon

**Affiliations:** 1 Department of Biochemistry, School of Medicine, Daegu Catholic University, Daegu, Republic of Korea; 2 Department of Biochemistry, College of Medicine, Soonchunhyang University, Cheonan, Republic of Korea; 3 Department of Anatomy, School of Medicine, Daegu Catholic University, Daegu, Republic of Korea; 4 Department of Neurology, School of Medicine, Daegu Catholic University, Daegu, Republic of Korea; 5 Department of Rehabilitation Medicine, School of Medicine, Daegu Catholic University, Daegu, Republic of Korea

**Keywords:** Alzheimer’s disease, donepezil, microcurrent therapy, amyloid-*β*, cognitive function, neuroinflammation, 5XFAD mouse model

## Abstract

**Background:**

Alzheimer’s disease (AD) is a neurodegenerative disorder due to Aβ plaque accumulation, followed by loss of synapses and decline in cognitive abilities. Donepezil is currently one of the standard pharmacological treatments for Alzheimer’s disease. Recently, microcurrent (MC) therapy has emerged as a non-pharmacological adjunct for AD management. Recently, microcurrent therapy emerged as a non-pharmacological alternative to treat AD.

**Objective:**

The study investigates the therapeutic outcomes of the MC as an adjuvant to donepezil in mitigating cognitive dysfunction in the transgenic mouse model (5XFAD).

**Methods:**

Transgenic 5xFAD mice were assigned to the control, donepezil, MC, or MC + donepezil (combination) groups. Behavioral performance was assessed using the novel object recognition (NOR) and radial arm maze (RAM) tests. Amyloid burden, glial activation, cytokine expression, apoptotic signaling, and intracellular pathways (PI3K–AKT, AMPK, and JAK2/3) were analyzed by immunohistochemistry and Western blotting.

**Results:**

Combined treatment with donepezil and microcurrent showed a trend toward improved cognitive performance and reduced pathology compared to donepezil alone, although these differences were not statistically significant. Aβ plaque burden in the cortex and the hippocampus was reduced by approximately 68%, thereby exceeding reductions observed with either treatment alone. Microglial and astroglial activation (Iba1, GFAP, and CD68) and pro-inflammatory cytokines (TNF-*α* and IL-1β) were reduced in both the donepezil and combination groups compared with untreated 5xFAD mice, with no significant difference between 5xD and 5xD + MC. Apoptotic markers (cleaved caspase-3 and cleaved PARP) were significantly reduced in both treatment groups compared with untreated controls but not significantly different between donepezil and combination therapy. At the molecular level, both donepezil and combination therapy activated PI3K–AKT and AMPK signaling and increased inhibitory phosphorylation of GSK-3β compared with untreated 5xFAD mice; no significant difference was observed between the two treatment groups.

**Conclusion:**

Donepezil combined with microcurrent therapy showed comparable efficacy to donepezil alone, with numerical trends toward further improvement in cognitive function and pathology, but without statistically significant differences. Both treatments reduced Aβ burden, attenuated glial activation, and modulated survival-related pathways to a similar extent. These findings support a multi-target therapeutic strategy and highlight the translational potential of integrating microcurrent therapy with standard pharmacological treatment for AD.

## Graphical abstract

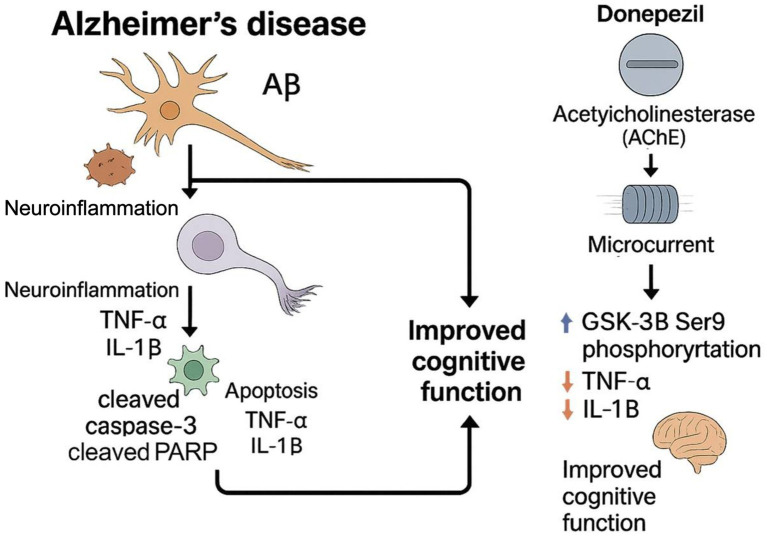

## Introduction

Alzheimer’s disease (AD) is one of the most common neurodegenerative disorders that affects the elderly aged above 65 years (Esiri and Chance, 2012). With over 35.6 million affected and a rate of 7.7 million new cases globally, AD remains one of the major causes of poor quality of life for the elderly (Pardo-Moreno et al., 2022). A loss of episodic memory attributable to cognitive impairments in the regions responsible for executive function, visuospatial ability, and others characterizes AD. Synaptic loss and cholinergic neuronal loss observed in AD are attributed to senile plaque depositions of parenchymal amyloid-*β* (Aβ) (Pardo-Moreno et al., 2022). Histologically, neuronal loss is often associated with neuroinflammation and neurodegeneration. Cholinesterase inhibitors such as donepezil are routinely used to treat AD (Kuwahata et al., 2021). However, several studies, such as those by Hecheng Wang, described that large doses of donepezil are essential to cause a significant change in cognitive function (Yang et al., 2013).

In recent years, several tissue engineering and regenerative medicine advancements have been studied. Microcurrent therapy has emerged as one of the non-invasive and impactful treatment protocols for AD-affected patients (Kong et al., 2024). The study by Li et al. highlighted the potential for axonal regeneration through Wallerian degeneration (Li et al., 2023). Nerve stimulation, when applied at a low intensity spectrum, has been accepted as a sustainable treatment option as a result of its low discomfort to patients (Hassan et al., 2020). A critical aspect of AD is the degeneration of tubulin proteins attributable to abnormalities in the tau protein, resulting in microtubular disintegration and disruption of axonal transport. Additionally, neuronal death is primarily caused by oxidative stress and mitochondrial dysfunction (Kim et al., 2024a).

Emerging evidence has shown that intracellular signaling pathways play a pivotal role in both neurodegeneration and therapeutic responses in AD (Pan et al., 2024). The PI3K–AKT axis is essential for neuronal survival and synaptic plasticity, mediating anti-apoptotic responses and regulating metabolism (Sun et al., 2019). AMPK, an energy-sensing kinase, promotes mitochondrial function and autophagic clearance, protecting cellular stress (Yang et al., 2020). Importantly, inactivation of GSK-3β through phosphorylation at Ser9 has been shown to reduce tau hyperphosphorylation and mitigate microtubule destabilization—two hallmarks of AD pathology (Medina and Avila, 2014). Additionally, the JAK2/3–STAT signaling pathway, beyond its classical role in cytokine-mediated immune responses, has been implicated in neuroimmune modulation and glial-driven repair in the CNS (Song and Suk, 2017). Activation of JAK2/3, when uncoupled from pro-inflammatory cytokine induction, may contribute to homeostatic or protective responses in neurodegenerative settings (Ha et al., 2022; Gabbouj et al., 2019).

Therefore, we propose that, while donepezil mitigates neuronal loss, microcurrent (MC) therapy may enhance regenerative signaling and restore intracellular homeostasis. The current study aims to evaluate the therapeutic efficacy of combined treatment with donepezil and MC therapy, focusing on their potential to activate PI3K–AKT–AMPK survival signaling, inhibit GSK-3β activity, and modulate JAK2/3-related immune signaling, ultimately inducing synergistic neuroprotection in AD.

## Materials and methods

### Chemicals and antibodies

The chemicals used in this study included the following: APP (Sigma-Aldrich, St. Louis, MO, USA); Iba-1 (ab178846, 1:1000, Abcam, Cambridge, UK); TGF; TNF-α (sc-52746, 1:1000, Santa Cruz Biotechnology, Dallas, TX, USA); IL-1β (sc-7884, 1:1000, Santa Cruz Biotechnology, Dallas, TX, USA); cresyl violet (C5042, Sigma-Aldrich, St. Louis, MO, USA); amyloid-β (sc-53822, 1:500, Santa Cruz Biotechnology, Dallas, TX, USA); β-actin (sc-8432, 1:1000, Santa Cruz Biotechnology, Dallas, TX, USA); Alexa Fluor 594 anti-mouse IgG (A11005, 1:1000, Invitrogen, Waltham, MA, USA); anti-goat rabbit IgG (ADI-SAB-300-J, 1:4000, Enzo Life Sciences, Farmingdale, NY, USA); anti-goat mouse IgG (ADI-SAB-100-J, 1:4000, Enzo Life Sciences, Farmingdale, NY, USA); ABC kit (PK-6100, Vector Laboratories, Newark, CA, USA); DAB kit (SK-4100, Vector Laboratories, Newark, CA, USA); and Alexa Fluor 488 anti-rabbit IgG (A32790, 1:1000, Invitrogen, Waltham, MA, USA).

### Microcurrent therapy

Microcurrent was applied at the set times and at the set dosage (Ecure, Buan, Republic of Korea). The electrical current passed through the cage floor and reached the mice’s brain tissues. Mice are nocturnal animals, and thus, the microcurrent is applied during their active time of the day. Kim et al. provided insight into the effectiveness of the stepform waveform in cognitive repair in AD mice, and thus, the same was applied (Kim et al., 2024b). A stepform waveform of 0, 1.7, 3.4, and 5 V was generated through wave superposition. The base frequency of 7 Hz with an extra 44 kHz frequency superimposition was set, along with the 5 V voltage and the microcurrent with a magnitude of 1 μA (500 ohms). This dosage was administered for 4 weeks in 6-h sessions.

### Animals

Mice (transgenic) with B6SJL Tg (APPSwFlLon, PSEN1*M146L* L286V) 6799Vas/Mmjax strain from Jackson Laboratory, located in Bar Harbor, ME, USA. Wild-type (WT) and Tg-5xFAD AD mice were divided into different groups and exposed to microcurrents at the age of 1.5 months. Genetic advancement of Aβ aggregation in Tg-5xFaD AD mice was initiated through this treatment. Permissions were obtained from the Institutional Animal Care and Use Committee, which granted permission to conduct these experiments (IRB no.: DCIAFCR-240613-13-Y), and the global standards for treating animals were adhered to. Post the 7-day adaptation period, a small group of Tg-5xFAD mice were randomly assigned to the AD model group or the microcurrent plus AD model group (*n* = 5 per group). Followed by further categorization into the + NC group (*n* = 5 per group) or donepezil, or donepezil + MC, or the normal control (NC) group, microcurrent therapy was initiated and was followed by behavioral analysis.

### Novel object recognition test (NOR)

The Novel Object Recognition test includes two objects, one that is familiar (TA) to the test group and the other unfamiliar (TB). Recognition memory is calculated using the discrimination index using the formula TB/(TA + TB) × 100. Mice were initially kept in a testing chamber at 23 ± 1 °C and 50–60% humidity with sufficient resources such as food and water throughout the night in the chamber. The mice are allowed to experience a 12-h dark–light cycle. During the training session, two circular filter units are kept inside the chamber, and these are of identical dimensions in height and diameter (27 and 33 mm, respectively). This is followed by an exploration session for 10 min. The following day, a 30 mm-long plastic cone of 25 mm diameter replaces the initially placed object. Object recognition is measured based on the time taken for identifying the object and for smelling or touching the newly placed item in a test period of 5 min (Antunes and Biala, 2012). The recording and analysis of the training and the assessment of the trials were conducted using EthoVision XT8.5 (Broadbent et al., 2010).

### Radial arm maze test (RAM)

Prior to and after microcurrent treatment, the Radial Arm Maze test was performed to assess the neurocognitive abilities, such as spatial operational memory, of the mice. The groups of mice included normal and non-Tg wild-type samples (Clark et al., 2015).

Prior to the assessment, mice underwent a 10-min training session in the radial arm maze. The radial arm contains a reward cup. During training days, the mice were permitted to explore the maze with a lure placed on each arm spaced 135 degrees apart. The performance was considered successful only when a mouse goes into the food-containing cup, whereas if it visits a cup without food or bait, it is considered a fail, suggesting an inaccurate spatial functional memory (Penley et al., 2013). The data were evaluated using an ANOVA to identify deviations.

### Tissue preparation

Animal tissue was prepared following a performed sacrifice as per the institutional policies and guidelines. Considering the statistical analysis validity, mice were assigned to each group randomly. Histological studies were conducted where the right hemisphere was stored at −80 °C overnight, while the left brain hemispheres were kept in a 4% paraformaldehyde solution. Hippocampal tissue was removed from the freezer −80 °C to perform Western blot analysis. The tissues from the hippocampal and entorhinal cortex regions of three mice were used for Western blotting assessment, in accordance with Paxinos and Franklin mapping (Paxinos and Franklin, 2019).

### Immunohistochemistry

Immunostaining was performed using the Vectastain Elite ABC kit bought from Vector Laboratories Inc., United States. Tissue sections were initially immersed in citrate buffer and boiled for 30 min. A methanol solution containing 0.3% H_2_O_2_ was used to inhibit peroxidase enzyme activity present in the tissues at room temperature (RT) for 15 min. This step was performed to prepare the sections for immunoperoxidase labeling. Sections were first blocked using horse serum and incubated at 4 °C with antibodies. These sections were further exposed to either mouse IgG or biotinylated goat anti-human antibodies at room temperature for 30 min. The avidin-biotin-based protein complex is involved in the immunoreaction at RT for 30 min. Peroxidase reaction was particularly performed using the DAB kit. In some reactions, the primary antibody was not used but was counterstained with Harris hematoxylin before mounting. The quantification of positive cell counts was performed by applying the ImageJ program (ver. 1.53a), a Java-based image processing software tool developed by the NIH in Bethesda, MD, USA.

### Analysis of Western blot

Radio-immunoprecipitation assay (RIPA) buffer (50 mM Tris–Cl, pH 7.4; 1% NP-40; 150 mM NaCl, and 1 mM EDTA) is used for extracting proteins, followed by the addition of protease inhibitors (1 mM PMSF, 1 μg/mL of aprotinin, 1 μg/mL of leupeptin, and 1 mM Na_3_VO_4_). Protein sample quantification is performed using Bradford and is further separated by size on SDS/polyacrylamide gel electrophoresis with a sample of 30 μg. Nitrocellulose membrane is used to transfer the separated proteins for identification, as described previously (Kim et al., 2019). *β*-actin was analyzed using identical protein samples rerun on separate gels, rather than by stripping and reprobing the same membranes, to ensure clear and independent detection of loading controls.

### Statistical analysis

Statistical analyses were conducted using a two-way ANOVA, followed by post-hoc analyses using Tukey’s multiple comparison test to determine specific group differences. Significance levels were set to *p* < 0.05, *p* < 0.01, and *p* < 0.001, denoted as *, **, and ***, respectively. All statistical computations were performed using GraphPad Prism, version 8.0.1.

## Results

### Combined effects of MC therapy combined donepezil on memory impairment in the 5xFAD AD mouse model

**Figure 1:**
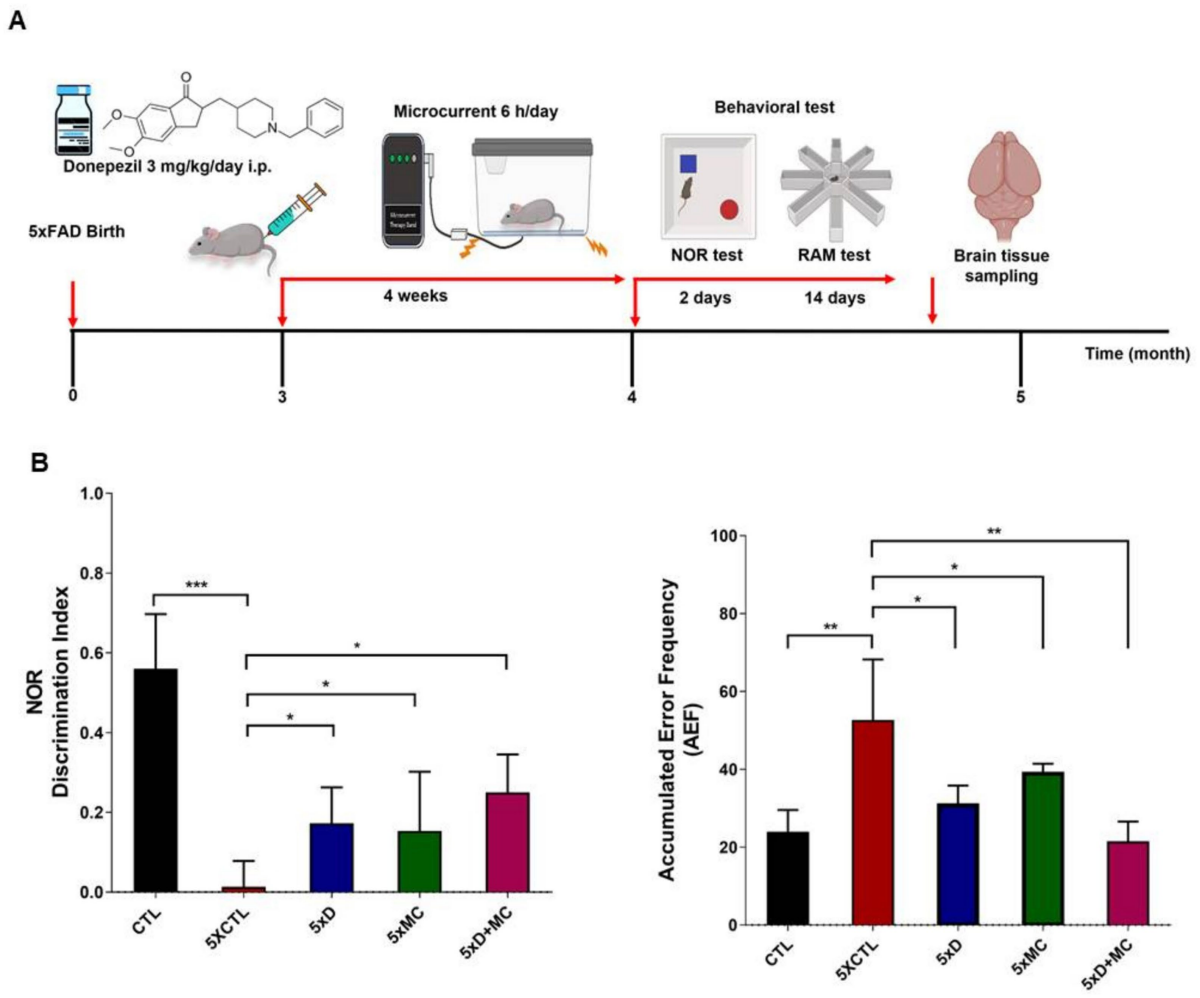
Donepezil combined with microcurrent therapy mitigates memory impairment. **(A)** An illustration of the experimental process. **(B)** The novel object recognition task was performed on 5xFAD mice (transgenic mice) and their control group (non-transgenic mice) (*n* = 5 per group). The discrimination index was computed by determining the percentage ratio of TB/(TA + TB) × 100, with TA referring to the known object and TB signifying the new object. The radial arm maze test was used to analyze spatial memory (CTL-untreated, 5xFAD-untreated, mc group, mc plus donepezil). *Columns,* mean of three independent experiments; *bars,* SD. *p < 0.05, **p < 0.01, ***p < 0.001.

5xFAD AD mice were used to examine whether MC therapy was capable of enhancing impaired memory. Figure 1A presents a brief outline of the research methodology. To study the percentage recognition index, the ratio of the time spent examining the novel object to the time when 5xFAD AD mice could identify familiar objects during the testing period was used. In comparison with the control group, the percentage recognition index increased substantially in the MC plus donepezil group compared with that of the single treatment. When the 5xFAD mice started recalling familiar objects more often, they became more intrigued by examining the novel object. Training session data were not recorded in this study, as behavioral assessments focused on the test phase to evaluate treatment effects. The MC + donepezil group showed numerically higher recognition memory performance compared with single-treatment groups; however, this difference was not statistically significant (Figures 1B,C).

### MC plus donepezil therapy reduced aβ levels and the amyloid burden within the cortex and the hippocampus of 5xFAD AD mice

**Figure 2:**
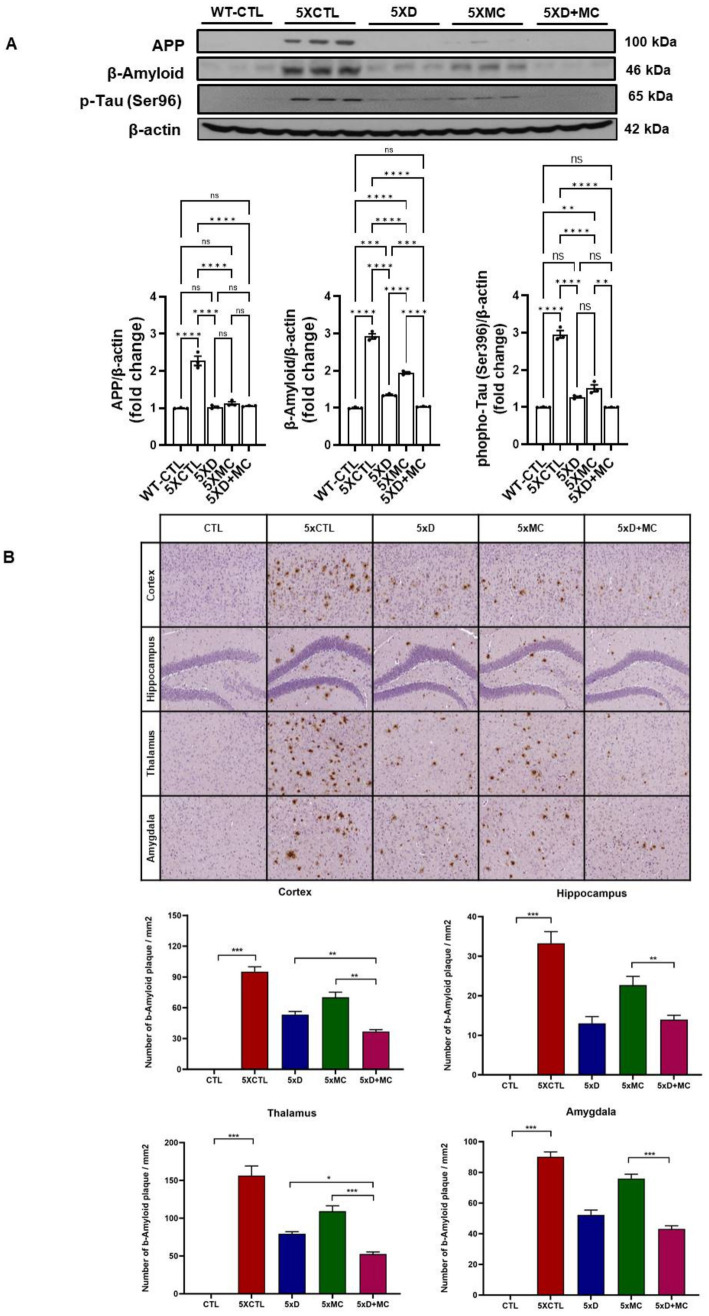
MC plus donepezil therapy reduced brain A*β* levels and improved the pathology of Aβ in AD mice compared with that of single treatment. **(A)** The quantification of APP, Aβ, and p-Tau expression by Western blotting was determined in each group. **(B)** The number of Aβ immunolabeled cells for each view and the extent of Aβ staining coverage in the brain of every group. *Columns,* mean of three independent experiments; *bars,* SD. *p < 0.05, **p < 0.01, ***p < 0.001.

The impact of MC therapy on Aβ pathology within the brain of the 5xFAD AD mice was studied using Aβ staining (Figures 2A,B) to examine the Aβ plaque. It was observed that, in the frontal cortex and the hippocampus regions, there was a low expression of Aβ-positive proteins of the control (CTL) group, while it was upregulated in the AD model group and inhibited in the group treated with MC plus donepezil treatment compared with that of a single treatment. The findings of the quantification analysis demonstrated that there was a substantial decrease in the amount of Aβ immunolabeled cells within the cortex and the hippocampus of the MC-treated group in comparison with the AD model group. The inhibition extent is much higher than that of single treatment. The regions covered with Aβ also exhibited a decrease of approximately 68% in the cortex and the hippocampus of the MC plus donepezil-treated group, in contrast to the single group.

### Attenuation of microglial and astroglial activation by donepezil and microcurrent therapy

**Figure 3:**
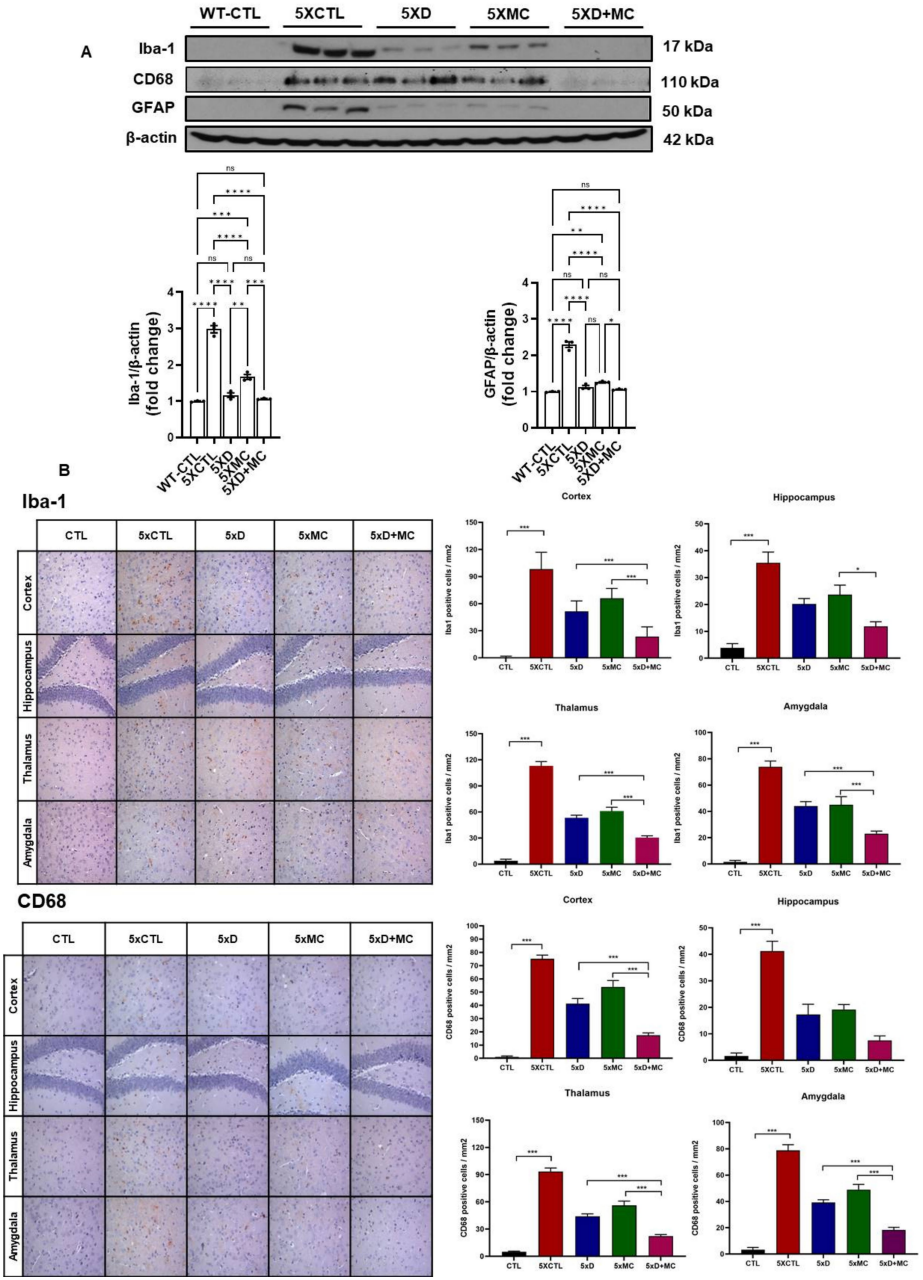
Attenuation of glial activation by donepezil and microcurrent therapy. **(A)** The quantification of Iba1, GFAP, and CD68 expression by Western blotting was determined in each group. **(B)** Immunohistochemical staining of Iba1, GFAP, and CD68 in the entorhinal cortex brain section of CTL and 5xFAD mice following microcurrent or donepezil therapy. *Columns,* mean of three independent experiments; *bars,* SD. *p < 0.05, **p < 0.01, ***p < 0.001.

To determine the situation of microglial cells in the brains of treated and normal mice, the amounts of Iba1 (as the marker of total microglia, including resting and activated states), astrocyte marker glial fibrillary acidic protein (GFAP), and CD68 (as the marker of activated microglia) were evaluated by Western blots. Increased Iba, GFAP, and CD68 expression, indicating heightened microglia activation, was evident in the 5xFAD mice when compared to the control group. (Figure 3A). Iba1, GFAP, and CD68 were reduced in both donepezil and combination groups compared with untreated 5xFAD mice, with no significant difference between 5xD and 5xD + MC (Figure 3B).

### Modulation of pro-inflammatory cytokine expression in the hippocampus by donepezil and microcurrent therapy

**Figure 4:**
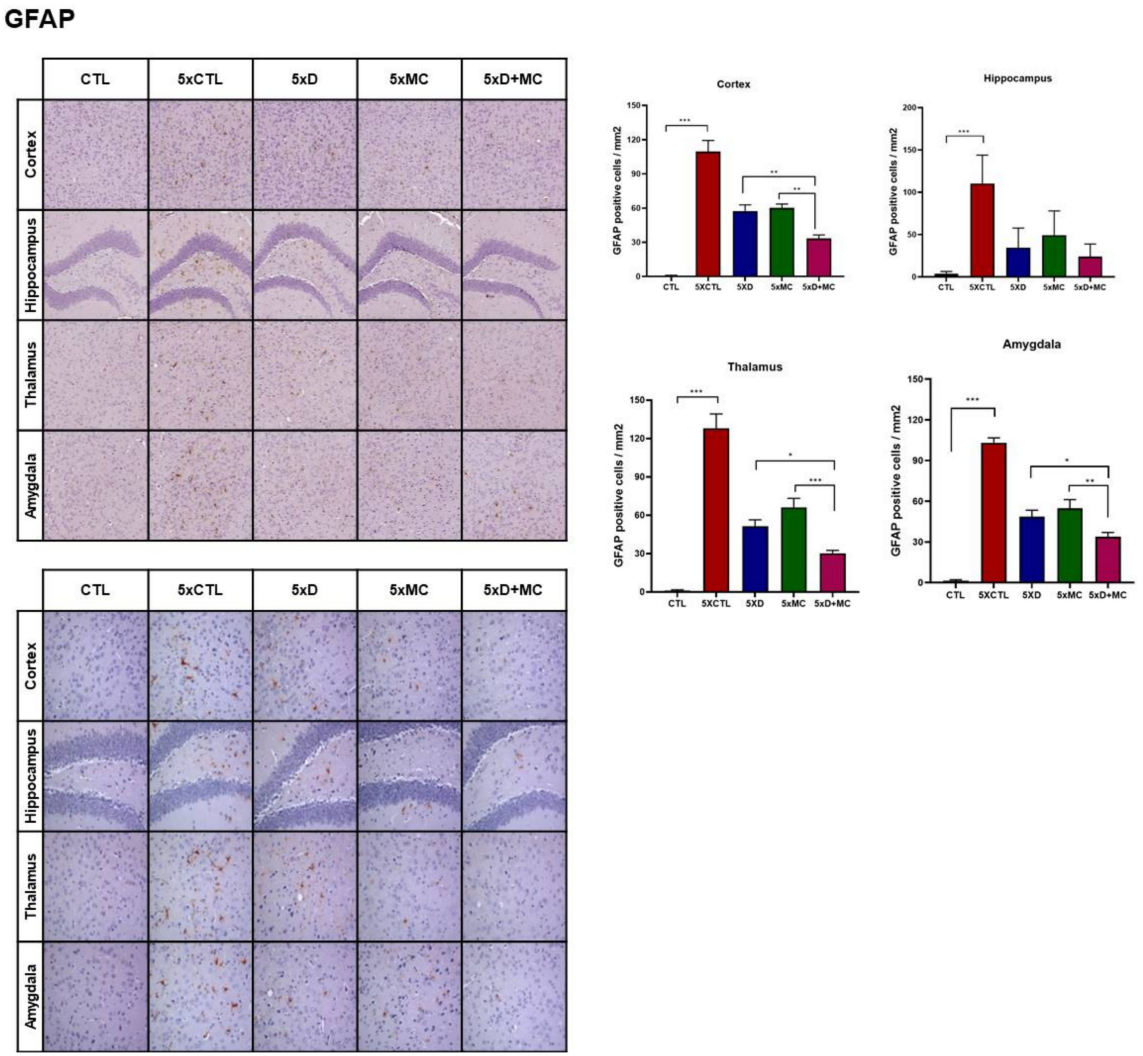
Modulation of cytokine expression by donepezil and microcurrent therapy. The quantification of IL-1β, TNF-*α*, and TGF-β expression by Western blotting was determined in each group. Western blot analyses of IL-1β, TNF-α, and TGF-β. AD mice showed reduced TNF-α and TGF-β. Donepezil or MC monotherapy partially normalized these alterations, whereas combination therapy more effectively suppressed TNF-α and restored TGF-β. *Columns,* mean of three independent experiments; *bars,* SD. *p < 0.05, **p < 0.01, ***p < 0.001.

To study neuroinflammation post-MC combined with donepezil treatment, we initially executed immunohistochemistry on the microglia marker. The inflammatory response in the transgenic mouse brain was quantitatively examined in the cortex and the hippocampus by evaluating the amount of the pro-inflammatory markers tumor necrosis factor *α* (TNFα) and interleukin-1β (IL-1β). Both donepezil and combination therapy reduced cytokine expression compared to untreated controls, but no additional reduction was observed with combination therapy compared to donepezil alone (Figure 4).

### Suppression of apoptotic signaling pathways by donepezil and microcurrent therapy in Alzheimer’s disease mice

**Figure 5:**
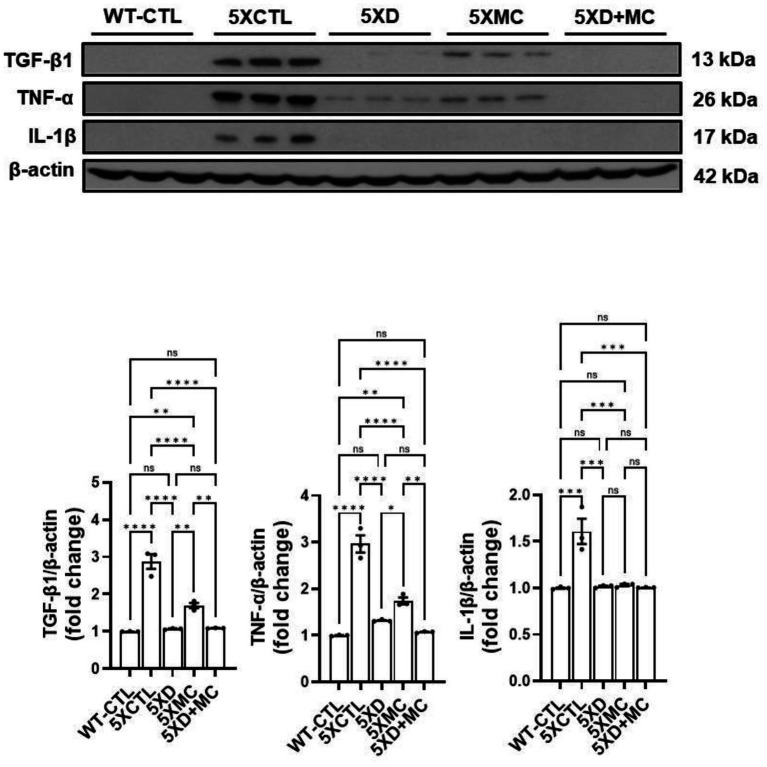
Suppression of apoptotic signaling pathways by donepezil and microcurrent therapy. The quantification of cleaved caspase-3 and cleaved PARP expression by Western blotting was determined in each group. Western blot analyses of cleaved caspase-3 and cleaved PARP. AD mice exhibited increased expression of both apoptotic markers compared with controls. Donepezil or MC monotherapy partially reduced these elevations, whereas combination therapy produced stronger suppression. *Columns,* mean of three independent experiments; *bars,* SD. *p < 0.05, **p < 0.01, ***p < 0.001.

To evaluate apoptosis-related changes, we measured the levels of cleaved caspase-3 and cleaved PARP in the hippocampus of 5xFAD mice (Figure 5). Both donepezil monotherapy and combination therapy (donepezil + MC) significantly reduced cleaved caspase-3 and cleaved PARP levels compared with the MC-only groups. However, no statistically significant difference was observed between the donepezil and combination groups. These findings suggest that the addition of microcurrent stimulation to donepezil did not further enhance the suppression of apoptosis markers and that both treatments exerted a comparable anti-apoptotic effect.

### Combination therapy modulates multiple intracellular signaling pathways related to survival, metabolism, and immune regulation

**Figure 6:**
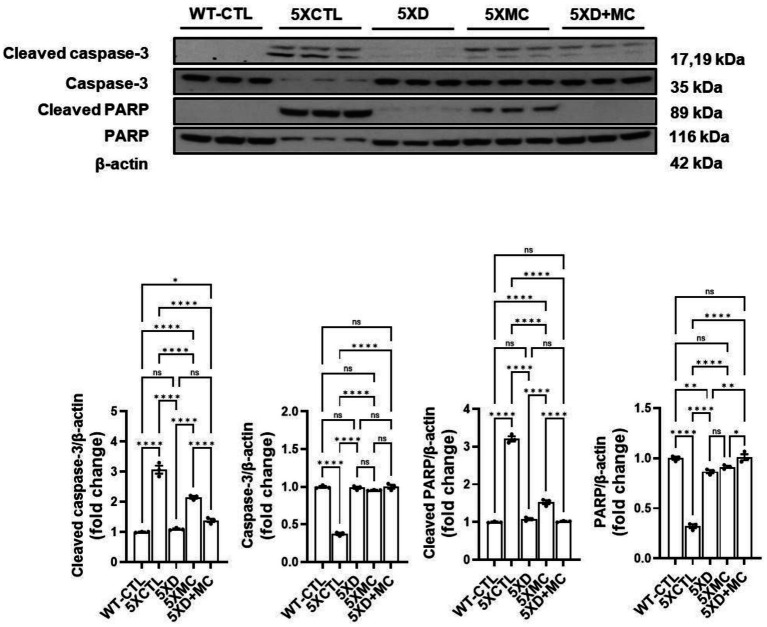
Combined donepezil and microcurrent therapy modulates key signaling pathways in 5xFAD mice. Representative Western blots and quantification of phosphorylated PI3K, AKT, AMPK, GSK-3β, JAK2, and JAK3 in hippocampal tissue from 5xFAD mice. Combination treatment significantly increased phosphorylation of all targets compared to single treatments or AD controls, indicating the activation of survival and immune-regulatory pathways. *Columns,* mean of three independent experiments; *bars,* SD. *p < 0.05, **p < 0.01, ***p < 0.001.

**Figure 7:**
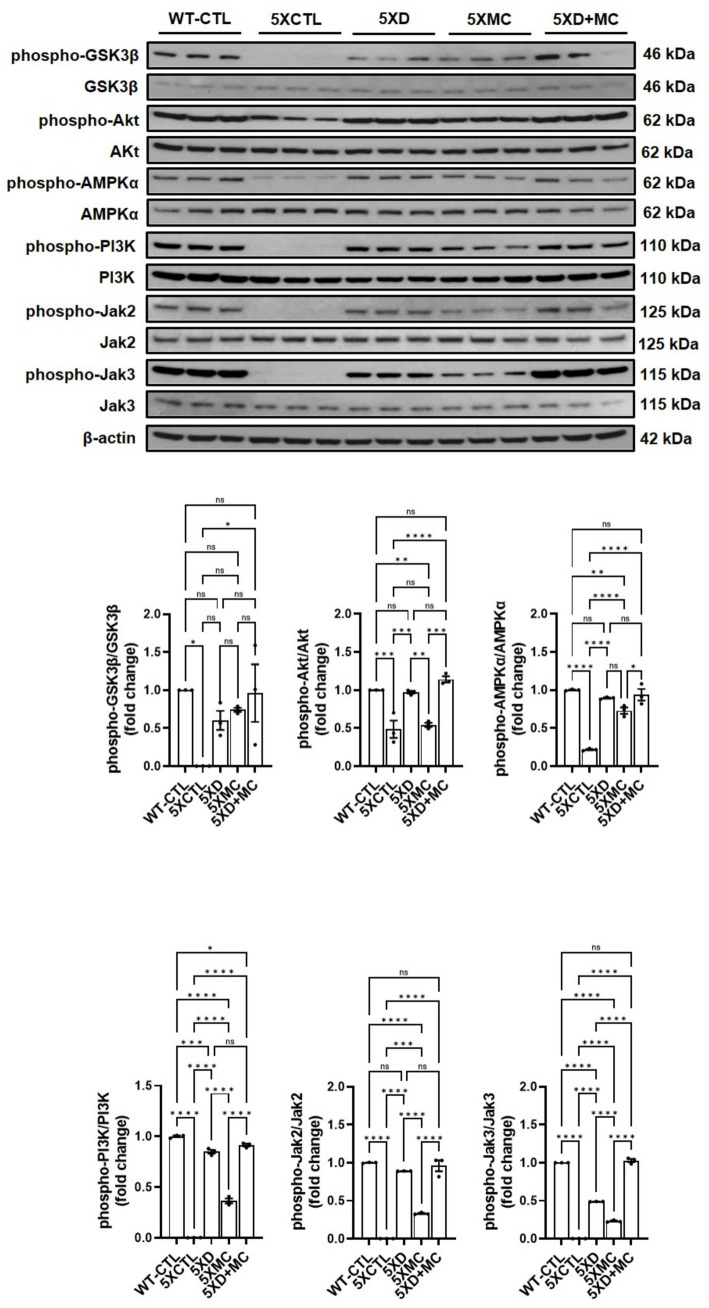
Schematic representation. A schematic summarizes the experimental workflow and key molecular and behavioral outcomes of combined microcurrent and donepezil therapy.

To explore the molecular mechanisms associated with the observed therapeutic effects, we analyzed the phosphorylation status of key intracellular signaling proteins in the hippocampus of 5xFAD mice. A Western blot analysis revealed that both donepezil and combination therapy significantly increased the phosphorylation of PI3K and AKT compared with the untreated and MC-only groups (Figure 6). However, no significant differences were observed between the combination and donepezil groups. Phosphorylation of AMPK was also significantly elevated in both treatment groups relative to controls, indicating activation of energy regulation pathways. In parallel, phosphorylated GSK-3β levels were increased to a similar extent in both groups, suggesting reduced GSK-3β activity and potential attenuation of tau-related pathology. Combination therapy also increased phosphorylated JAK2 and JAK3 compared with controls, but levels were not significantly different from donepezil alone, while pro-inflammatory cytokine levels (TNF-*α* and IL-1β) remained unchanged or slightly reduced. A graphical scheme was added to summarize the workflow and major outcomes of the present research (Figure 7). These findings indicate that both treatments activated PI3K–AKT, AMPK, and JAK2/3 signaling pathways and inhibited GSK-3β, which may contribute to neuronal survival, metabolic regulation, and immune homeostasis in AD pathology.

## Discussion

The current study showed donepezil treatment with microcurrent therapy investigated the effect on cognitive function in AD patients. The results indicate an overall improvement in Aβ plaque burden and memory impairment. The radial arm maze (RAM) and novel object recognition (NOR) assessments aim at the cognitive function of the treatment. The combination of donepezil and MC therapy showed a trend toward reduced error rates and improved spatial memory compared with monotherapies, although these differences were not statistically significant in this pilot study. These results indicate an improvement in spatial memory. Furthermore, the NOR assessment showed prominent object recognition in the combination-treated mice compared to those treated individually with either MC therapy or donepezil alone (Birks and Harvey, 2018). Our findings are consistent with this report, which supports the individual efficacy of donepezil (Birks and Harvey, 2018) and the neuroprotective potential of low-intensity electrical stimulation (Kong et al., 2024; Li et al., 2023). This study suggests a potential additive trend in combining these treatments in a transgenic AD model, which may be attributed to distinct but complementary mechanisms: donepezil primarily augments cholinergic transmission, whereas MC therapy influences neuroinflammation, synaptic plasticity, and cellular repair.

Aβ plaque burden is a common occurrence among AD patients (Kellner et al., 2009). An immunohistochemical analysis of the hippocampal region in the 5xFAD mice showed a decline in the Aβ plaque region of up to 68% among the MC plus donepezil-treated 5xFAD mice. Given the role of Aβ plaques in AD pathogenesis, the reduction is strongly associated with the prevention of cognitive decline (Zhang and Jiang, 2015). On the other hand, the MC-single-treated mice were found to have a 59% reduction in the plaque-affected region. The Western blotting analysis was used to assess pro-inflammatory markers, such as cytokines and interleukin-1 beta (IL-1β), particularly tumor necrosis factor-alpha (TNF-*α*), in both the hippocampus and cortex regions. The expression of the inflammatory receptors is crucial to determine ongoing processes, as these modulate the immune responses in the tissues. The study results reveal a significant reduction in inflammation, a prominent sign of neuroprotection.

Both donepezil and combination therapy activated PI3K–AKT, AMPK, and JAK2/3 signaling and increased inhibitory phosphorylation of GSK-3β compared with controls. However, no significant differences were observed between the combination and donepezil groups, indicating that microcurrent did not further enhance these signaling effects in this study. Elevated phosphorylation of PI3K and AKT indicates the potentiation of the canonical survival pathway, whereas increased phosphorylation of AMPK suggests enhanced metabolic resilience and mitochondrial function. These findings align with and extend previous reports. Pan et al. (2024) demonstrated AKT-mediated rescue of synaptic integrity in AD models, while Yang et al. (2020) highlighted AMPK activation via metabolic modulators. However, neither study incorporated combinatorial paradigms nor assessed glial regulatory cascades. Similarly, Li et al. (2023) and Kong et al. (2024) reported MC-induced neuroregeneration and anti-inflammatory effects, yet they did not integrate these effects with pharmacological interventions or provide deep molecular profiling. Our study uniquely integrates these modalities, offering a multi-axis therapeutic mechanism that simultaneously targets amyloid deposition, neuroinflammation, synaptic plasticity, and glial modulation.

These findings suggest that microcurrent may serve as an adjunctive approach that complements donepezil therapy by supporting neuronal repair and immune regulation, although no statistically significant synergistic effect was observed. This finding aligns with recent reviews proposing that multi-target strategies are essential for addressing the multifactorial pathology of AD (Yamada and Iwatsubo, 2024; Zhang and Jiang, 2015).

These results not only show the emerging role of MC therapy in CNS disorders but also provide a strong rationale for further translational research into combination treatment strategies. This study is limited by its small sample size (*n* = 5 per group) as a pilot study, which may affect the statistical value and generalizability of the results. Accordingly, future large-scale and longitudinal studies are warranted to validate these preliminary results and further define the therapeutic potential of the combined intervention. Future clinical trials would be needed to consider long-term efficacy, safety, and optimal stimulation parameters in AD patients to evaluate the persistence of therapeutic effects and potential side effects after combination treatment. Our study demonstrates significant changes at the behavioral and molecular levels; the precise mechanisms by which MC therapy exerts its anti-amyloid and anti-inflammatory effects remain to be elucidated.

## Data Availability

The original contributions presented in the study are included in the article and its Supplementary material. Further inquiries can be directed to the corresponding author.

## References

[ref1] AntunesM. BialaG. (2012). The novel object recognition memory: neurobiology, test procedure, and its modifications. Cogn. Process. 13, 93–110. doi: 10.1007/s10339-011-0430-z, PMID: 22160349 PMC3332351

[ref2] BirksJ. S. HarveyR. J. (2018). Donepezil for dementia due to Alzheimer's disease. Cochrane Database Syst. Rev. 6:Cd001190. doi: 10.1002/14651858.Cd001190.pub3, PMID: 12917900

[ref3] BroadbentN. J. GaskinS. SquireL. R. ClarkR. E. (2010). Object recognition memory and the rodent hippocampus. Learn. Mem. 17, 5–11. doi: 10.1101/lm.1650110, PMID: 20028732 PMC2807177

[ref4] ClarkJ. K. FurgersonM. CrystalJ. D. FechheimerM. FurukawaR. WagnerJ. J. (2015). Alterations in synaptic plasticity coincide with deficits in spatial working memory in presymptomatic 3xTg-ad mice. Neurobiol. Learn. Mem. 125, 152–162. doi: 10.1016/j.nlm.2015.09.003, PMID: 26385257 PMC4648633

[ref9001] EsiriM. M. ChanceS. A. (2012). Cognitive reserve, cortical plasticity and resistance to Alzheimer’s disease. Alzheimer’s Res. Ther, 4:7. doi: 10.1186/alzrt10522380508 PMC3334540

[ref5] GabboujS. RyhänenS. MarttinenM. WittrahmR. TakaloM. KemppainenS. . (2019). Altered insulin signaling in Alzheimer's disease brain - special emphasis on Pi3K-Akt pathway. Front. Neurosci. 13:629. doi: 10.3389/fnins.2019.00629, PMID: 31275108 PMC6591470

[ref6] HaG. H. KimE. J. ParkJ. S. KimJ. E. NamH. YeonJ. Y. . (2022). Jak2/Stat3 pathway mediates neuroprotective and pro-angiogenic treatment effects of adult human neural stem cells in middle cerebral artery occlusion stroke animal models. Aging (Albany NY) 14, 8944–8969. doi: 10.18632/aging.204410, PMID: 36446389 PMC9740376

[ref9004] HassanA. RobinsonM. WillerthS. M. (2020). Determining the mechanism behind yoga’s effects on preventing the symptoms of Alzheimer’s disease. Neural Regen. Res, 15, 261–262. doi: 10.4103/1673-5374.26555331552895 PMC6905332

[ref8] KellnerA. MatschkeJ. BernreutherC. MochH. FerrerI. GlatzelM. (2009). Autoantibodies against beta-amyloid are common in Alzheimer's disease and help control plaque burden. Ann. Neurol. 65, 24–31. doi: 10.1002/ana.21475, PMID: 19194878

[ref9] KimE. H. JoY. SaiS. ParkM. J. KimJ. Y. KimJ. S. . (2019). Tumor-treating fields induce autophagy by blocking the Akt2/miR29b axis in glioblastoma cells. Oncogene 38, 6630–6646. doi: 10.1038/s41388-019-0882-7, PMID: 31375748

[ref10] KimE. H. LeeW. S. LeeJ. H. KwonD. R. (2024a). Microcurrent therapy as the nonpharmacological new protocol against Alzheimer's disease. Front. Aging Neurosci. 16:1344072. doi: 10.3389/fnagi.2024.1344072, PMID: 38304741 PMC10833500

[ref9007] KimE. H. LeeW. S. KwonD. R. (2024b). Microcurrent therapy mitigates neuronal damage and cognitive decline in an Alzheimer’s disease mouse model: insights into mechanisms and therapeutic potential. Int. J. Mol. Sci. 25:6088. doi: 10.3390/ijms2511608838892278 PMC11173257

[ref11] Kong J. Teng C. Liu F. Wang X. Zhou Y. Zong Y. . (2024). Enhancing regeneration and repair of long-distance peripheral nerve defect injuries with continuous microcurrent electrical nerve stimulation. Front. Neurosci. 10:181361590. doi: 10.3389/fnins.2024.1361590 PMC10885699 38406586

[ref9002] Kuwahata S. Takenaka T. Motoya T. Masuda K. Yonezawa H. Shinchi S. . (2021). Effect of QT Prolongation in Patients Taking Cholinesterase Inhibitors (Donepezil) for Alzheimer’s Disease. Circulation Reports, 33, 115–121. doi: 10.1253/circrep.CR-20-0115 PMC7956884 33738343

[ref12] LiX. ZhangT. LiC. XuW. GuanY. LiX. . (2023). Electrical stimulation accelerates Wallerian degeneration and promotes nerve regeneration after sciatic nerve injury. Glia 71, 758–774. doi: 10.1002/glia.24309, PMID: 36484493

[ref13] MedinaM. AvilaJ. (2014). New insights into the role of glycogen synthase kinase-3 in Alzheimer's disease. Expert Opin. Ther. Targets 18, 69–77. doi: 10.1517/14728222.2013.843670, PMID: 24099155

[ref9006] PanJ. YaoQ. WangY. ChangS. LiC. WuY. . (2024). The role of PI3K signaling pathway in Alzheimer,s disease. Front. Aging Neurosci, 16:1459025. doi: 10.3389/fnagi.2024.145902539399315 PMC11466886

[ref9003] Pardo-MorenoT. González-AcedoA. Rivas-DomínguezA. García-MoralesV. García-CozarF. J. Ramos-RodríguezJ. J. . (2022). Therapeutic Approach to Alzheimer’s Disease: Current Treatments and New Perspectives. Pharmaceutics, 14:1117. doi: 10.3390/pharmaceutics1406111735745693 PMC9228613

[ref14] Paxinos G. Franklin K. B. (2019). Paxinos and Franklin’s the mouse brain in stereotaxic coordinates, compact: The coronal plates and diagrams. Cambridge, MA: Academic Press.

[ref15] Penley S. C. Gaudet C. M. Threlkeld S. W. (2013). Use of an eight-arm radial water maze to assess working and reference memory following neonatal brain injury. Journal of Visualized Experiments, 82:50940. doi: 10.3791/50940 PMC4030456 24335781

[ref16] Song G. J. Suk K. (2017). Jak–Stat signaling in neuroinflammation and Alzheimer’s disease. Jak-Stat 6:e1296151. doi: 10.1080/21623996.2017.1296151

[ref17] SunP. YinJ.-B. LiuL.-H. GuoJ. WangS.-H. QuC.-H. . (2019). Protective role of dihydromyricetin in Alzheimer’s disease rat model associated with activating Ampk/Sirt1 signaling pathway. Biosci. Rep. 39:Bsr20180902. doi: 10.1042/BSR20180902, PMID: 30498091 PMC6328867

[ref18] YamadaK. IwatsuboT. (2024). Involvement of the glymphatic/meningeal lymphatic system in Alzheimer's disease: insights into proteostasis and future directions. Cell. Mol. Life Sci. 81:192. doi: 10.1007/s00018-024-05225-z, PMID: 38652179 PMC11039514

[ref1101] Yang L. Bu L. Sun W. Li W. Liu J. Wu J. . (2020). AMPK: potential therapeutic target for Alzheimer’s disease. Curr. Protein Pept. Sci., 21, 66–77. doi: 10.2174/1389203720666190819142746 31424367

[ref1102] Yang Y.-H. Chen C.-H. Chou M.-C. Li C.-H. Liu C.-K. Chen S.-H. (2013). Concentration of donepezil to the cognitive response in Alzheimer disease. Clin. Psychopharmacol, 33, 351–355. doi: 10.1097/JCP.0b013e31828b5087 23609381

[ref19] ZhangF. JiangL. (2015). Neuroinflammation in Alzheimer's disease. Neuropsychiatr. Dis. Treat. 11:243–56. doi: 10.2147/ndt.S75546, PMID: 25673992 PMC4321665

