# Supplementary figures and images for: Evaluation of microcurrent as an adjunct to donepezil therapy in an Alzheimer’s disease mouse model: a pilot study
Source: Front Aging Neurosci. 2025 Nov 27;17:1689593. doi: 10.3389/fnagi.2025.1689593 (PMC12695830; doi:10.3389/fnagi.2025.1689593)

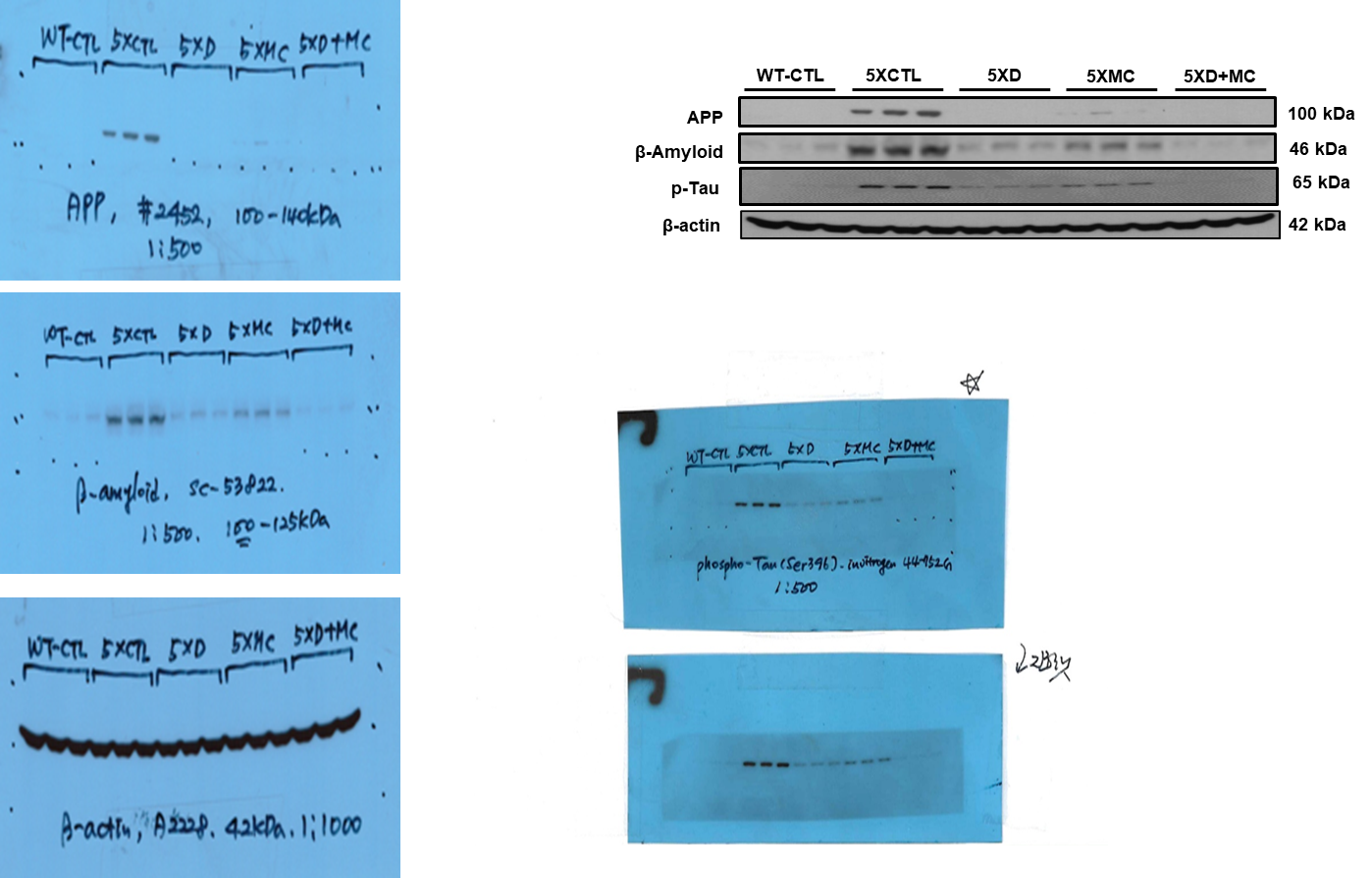

Supplement: Supplementary file 1 [file Image_1.TIF]

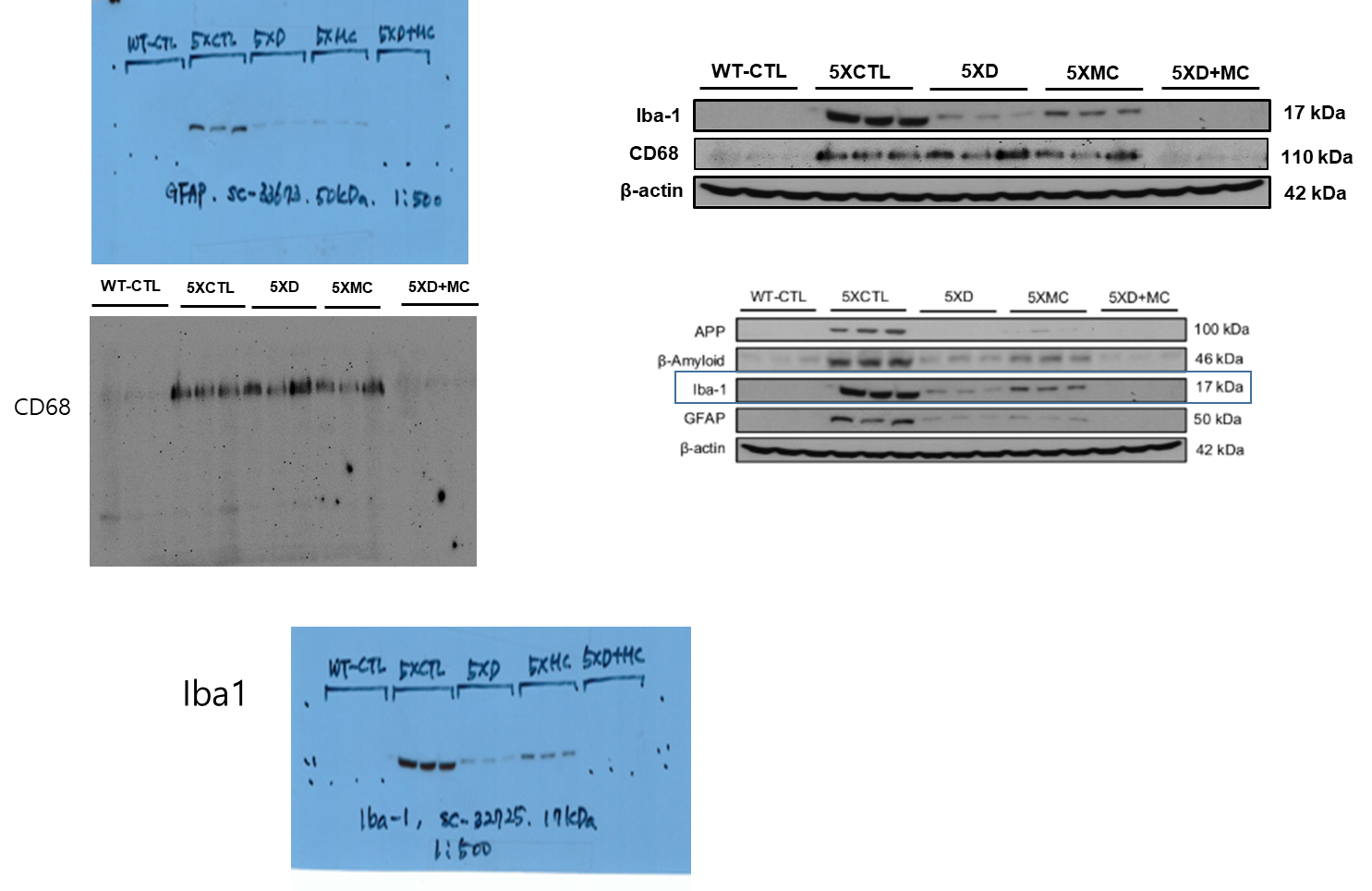

Supplement: Supplementary file 2 [file Image_2.TIF]

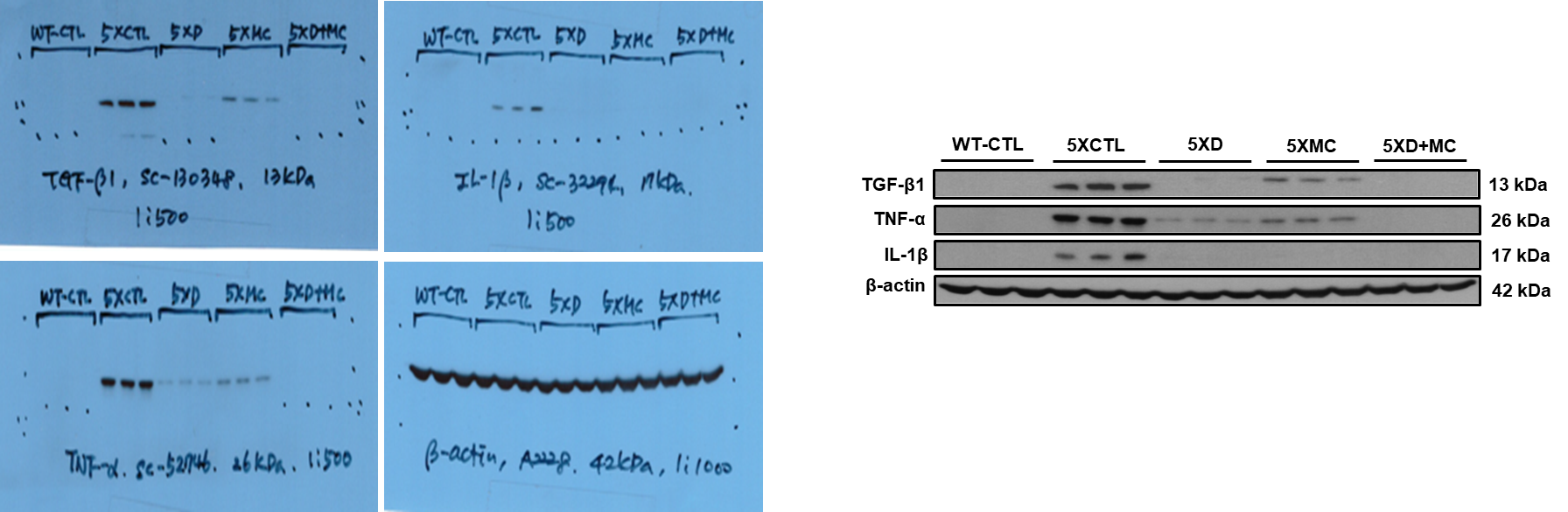

Supplement: Supplementary file 3 [file Image_3.TIF]

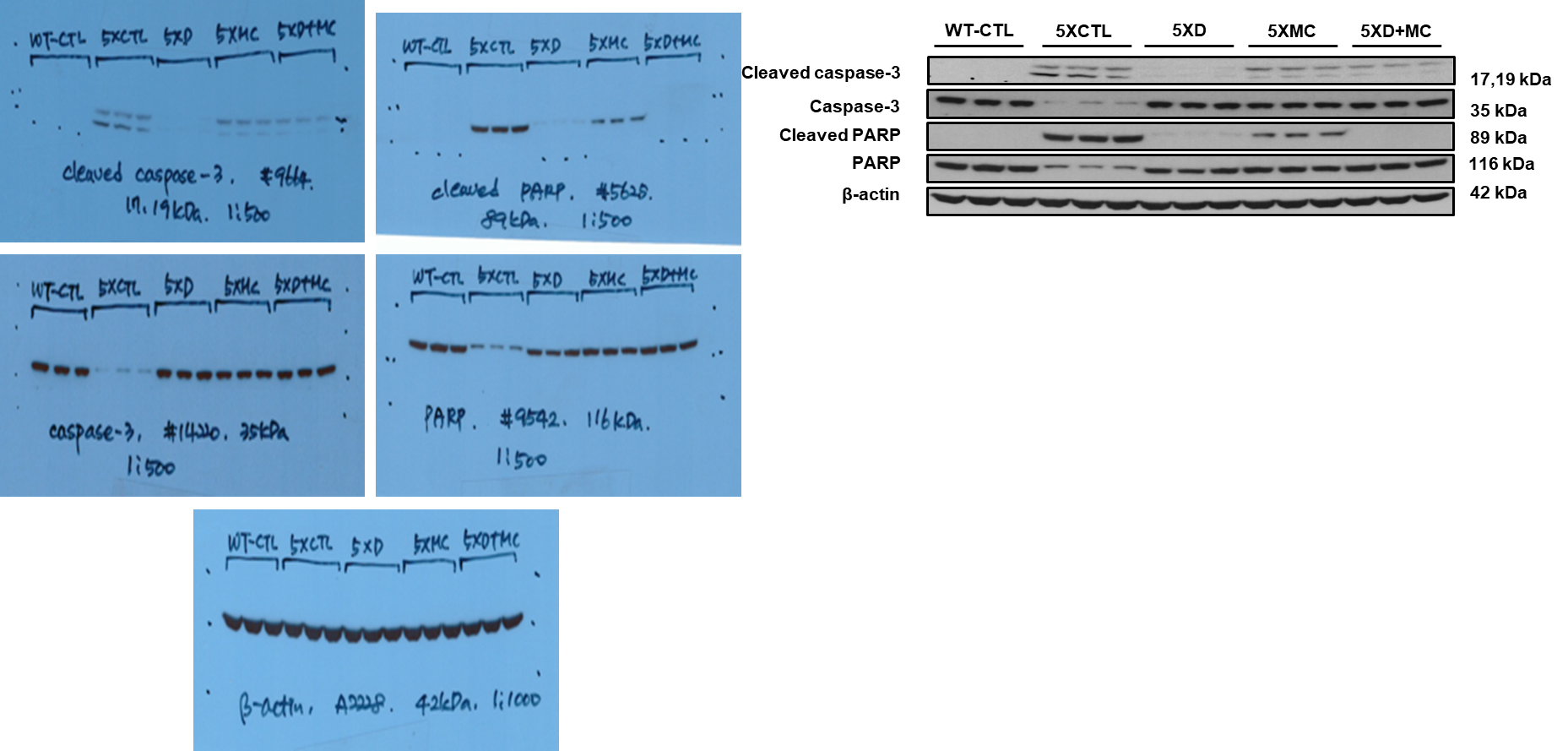

Supplement: Supplementary file 4 [file Image_4.TIF]
